# Comparative outcomes of spinal cord stimulation for neuropathic pain: peripheral nerve versus spinal cord lesions

**DOI:** 10.1007/s10143-025-03950-y

**Published:** 2026-01-10

**Authors:** Diana Noma Fitzrol, Bunpot Sitthinamsuwan, Sukunya Jirachaipitak, Pramote Euasobhon, Nantthasorn Zinboonyahgoon, Sarun Nunta-aree

**Affiliations:** 1 Division of Neurosurgery, Department of Surgery, Faculty of Medicine Siriraj Hospital, Mahidol University, 2 Wang Lang Road, Bangkok Noi, Bangkok, 10700 Thailand; 2 Department of Neurosciences, School of Medical Sciences, Universiti Sains Malaysia, Kelantan, Malaysia; 3 Department of Anesthesiology, Faculty of Medicine Siriraj Hospital, Mahidol University, Bangkok, Thailand; 4 Siriraj Pain Management Unit, Faculty of Medicine Siriraj Hospital, Mahidol University, Bangkok, Thailand

**Keywords:** Comparison, Neuropathic pain, Outcome, Peripheral nerve, Spinal cord, Spinal cord stimulation

## Abstract

Spinal cord stimulation (SCS) is commonly used to treat refractory neuropathic pain. However, no prior study has compared SCS outcomes between neuropathic pain of peripheral nerve origin and neuropathic pain of spinal cord origin. This study aimed to compare SCS outcomes between these two patient groups. Twenty-seven patients with refractory neuropathic pain underwent SCS. Of these, 14 had peripheral nerve lesions, whereas 13 had spinal cord lesions. Demographic data, numeric pain rating scores, and SCS outcomes were collected. These parameters were then compared between the two groups. Patients with neuropathic pain of peripheral nerve origin had more localized pain distribution (*p* = 0.012, OR = 8.33, 95% CI = 1.47‒47.23) than those with spinal cord origin. They also had a higher rate of successful trial stimulation (*p* = 0.018, OR = 9.60, 95% CI = 1.48‒62.16) and better long-term pain relief (*p* = 0.006). The poorest outcomes were observed in those with traumatic spinal cord injury. No complications occurred in this series. Patients harboring neuropathic pain of peripheral nerve origin demonstrated a higher success rate of trial stimulation and superior long-term outcomes following SCS compared with patients who had spinal cord lesions. These findings may aid clinicians in forecasting SCS outcomes and selecting appropriate candidates for the procedure.

## Introduction

Neuropathic pain arises from a lesion or disease affecting the somatosensory system [1, 2]. It can result from disorders involving the peripheral nerve, nerve plexus, spinal nerve root, spinal cord, or brain. Its prevalence ranges from 3% to 10% in the general population, creating a significant healthcare burden [3–6]. Chronic neuropathic pain also diminishes quality of life, imposes stress on caregivers, and generates a substantial economic burden [6, 7]. In contrast, effective pain control has been linked to improvements in patients’ physical, mental, and emotional well-being.

Although the exact mechanisms underlying neuropathic pain remain uncertain, both peripheral and central processes likely contribute. Injury to the first-order neuron may lower the depolarization threshold and increase ectopic discharges, thus triggering spontaneous pain. At the dorsal horn or brainstem, damage to inhibitory pathways can further facilitate pain. Moreover, the sympathetic nervous system may play a key role in its pathogenesis [8].

The Gate Control Theory proposed by Melzack and Wall in 1965 led to the emergence of spinal cord stimulation (SCS) as a treatment option for chronic intractable pain [9]. Although the precise mechanism of SCS in pain control is complex, its analgesic efficacy has been well documented [10–12]. SCS modulates nociceptive transmission at both the spinal and supraspinal levels via ascending and descending pathways [13]. At the cellular and molecular levels, glial cells and the balance of pro- and anti-inflammatory mediators may play roles in SCS-mediated pain modulation [14, 15]. Currently, SCS is considered beneficial for patients with refractory chronic neuropathic pain of the trunk and extremities [16–20], and it has proven to be both clinically effective and cost-effective in these populations [21].

Indications for SCS traditionally include failed back surgery syndrome, complex regional pain syndrome, and ischemic pain from peripheral vascular disease [22]. Neuropathic pain stemming from various other etiologies is less commonly described as an indication. Although several studies have examined SCS for chronic neuropathic pain, no previous investigation has compared outcomes between patients whose neuropathic pain originates in the peripheral nerve and those whose lesion is located in the spinal cord. Therefore, we conducted this study to elucidate differences in therapeutic outcomes of SCS between these two groups.

## Materials and methods

### Patient population

This study included patients treated with SCS at our medical center between June 2016 and February 2024. Eligible patients had intractable neuropathic pain caused by primary lesions either in the peripheral nerve or in the spinal cord. All patients initially underwent a 1‒2-week SCS trial.

The trial results were classified as either “positive” or “negative.” A positive trial was defined as a ≥ 50% reduction in neuropathic pain compared with preoperative baseline. Patients who met this criterion proceeded to permanent spinal cord stimulator implantation. In contrast, those who did not achieve ≥ 50% pain reduction were classified as having a negative trial and did not receive the device.

The exclusion criteria were (1) pain not primarily caused by peripheral nerve or spinal cord lesions, (2) coexisting peripheral nerve and spinal cord lesions in an individual patient, (3) multifocal neural lesions of uncertain origin, (4) failed back surgery syndrome, (5) failed neck surgery syndrome, (6) complex regional pain syndrome type I or II, and (7) ischemic pain due to peripheral vascular disease.

### Ethics approval

The Siriraj Institutional Review Board, Faculty of Medicine Siriraj Hospital, Mahidol University, Bangkok, Thailand, approved this study (reference number: Si 378/2024). All research procedures conformed to the 1964 Declaration of Helsinki and its later amendments or comparable ethical standards. Patient data remained confidential in accordance with these guidelines.

### Preoperative evaluation and operative procedures

At our institution, patients with intractable neuropathic pain who were potential SCS candidates underwent comprehensive assessments by a pain specialist, a neurosurgeon, and a psychiatrist. Major contraindications for SCS, such as active psychiatric illness or unrealistic outcome expectations, precluded progression to the trial stimulation.

Once deemed suitable, patients received detailed information about the SCS process, including trial electrode placement, trial stimulation, trial outcome assessment, and possible device implantation. Target spinal levels and sides for stimulation were determined by each patient’s pain distribution.

### Trial electrode placement

This procedure was conducted in a sterile operating room, with patients awake and positioned prone. Under intraoperative fluoroscopy, the interlaminar space for epidural access and the appropriate skin entry site were identified. Local anesthetic was administered at the puncture site and into the deeper fascia and muscles.

A 14-gauge, 3.5-inch Tuohy epidural needle was inserted in a superomedial direction, and the epidural space was confirmed using the loss-of-resistance technique. An 8-contact percutaneous trial electrode (Vectris 1 × 8 percutaneous lead, Medtronic, Fridley, MN, United States) was then introduced through the needle into the epidural space. Its position was verified fluoroscopically in anteroposterior and lateral views. In cases of misdirection, the electrode was repositioned immediately. If necessary, a second electrode was placed by following the same procedure. The electrode was positioned at the spinal cord segments that aligned with each patient’s specific pain distribution. In individuals with neuropathic pain resulting from spinal cord lesion, the lead site may be on the lesion, below the lesion, or above the lesion, based on the pain distribution for individual patient.

After guiding the electrode(s) to the targeted vertebral levels, they were connected to an external neurostimulator. The connectivity and impedance of the SCS system were confirmed, and the electrode contacts and stimulation parameters were adjusted intraoperatively to ensure comprehensive coverage of the painful area. Patients provided real-time feedback about pain coverage. Once appropriate coverage was achieved, the electrodes were anchored to the skin to prevent migration. If adequate coverage remained elusive, the electrode location and/or stimulation parameters were revised until the pain area was fully covered. The surgical site was then protected with a waterproof dressing.

Following the procedure, the external stimulator was switched on, and further adjustments to the stimulus parameters and programming were made to attain effective pain relief. Tonic stimulation, a traditional method for treating chronic neuropathic pain, was utilized. We used constant, low-frequency (40–60 Hz) electrical pulses to create a constant tingling sensation (paresthesia) that masks the pain. The stimulus parameters, including various active electrode contacts, electric current intensity, frequency, and pulse width were tailored to an individual patient. The stimulus parameters and stimulation programs were carefully adjusted to maximize pain relief. Most patients were admitted for 2 to 3 days for optimization of these settings before discharge.

### Trial stimulation phase

During the trial phase, patients were encouraged to resume normal daily activities while the stimulator was active, with an emphasis on avoiding prolonged sedentary behavior. They could adjust stimulation programs based on personal preference and pain relief outcomes. The trial period typically lasted 7‒10 days and did not exceed 14 days. All trial electrodes were later removed in the outpatient department.

### Outcome assessment of the trial stimulation

During follow-up visits, the impact of SCS trial stimulation was thoroughly evaluated, including pain reduction, mood, and performance in daily activities. Patients who experienced significant pain relief (i.e., ≥ 50% reduction in pain) were classified as having a “positive” trial and were considered candidates for permanent SCS device implantation.

### Implantation of the SCS device

The permanent device was typically implanted at least 4 weeks after the trial phase. Various electrode leads were used, including 8-contact percutaneous leads (Vectris SureScan MRI 1 × 8 percutaneous lead, Medtronic) and 16-contact surgical leads (Specify 5‒6‒5 or Specify 2 × 8, Medtronic). Most patients received a percutaneous lead, whereas a minority required a 2 × 8 surgical lead for cervical cord stimulation or a 5‒6‒5 surgical lead for thoracic or lumbosacral cord stimulation. The 8-contact percutaneous lead was utilized in instances without prior spinal surgery or in cases with previous spinal surgery where trial lead placement was feasible (not hindered by epidural fibrosis). The surgical lead types were chosen in situations where prior laminectomy had resulted in significant epidural fibrosis, complicating or preventing percutaneous lead placement. The insertion technique for percutaneous leads was comparable to that used during the trial phase, whereas open surgery was required in surgical lead placement. The final electrode positioning was centered on the vertebral levels where maximal pain relief was achieved.

Once optimal positioning was confirmed intraoperatively, total intravenous anesthesia was administered. A subcutaneous pocket for the implantable pulse generator (IPG) was created in the lateral back, and the electrode leads were anchored to adjacent fascia. A strain relief loop of the electrode wires was then formed, and the leads were passed through a subcutaneous tunnel and connected to the IPG. Both rechargeable IPG models (Intellis with AdaptiveStim technology and RestoreSensor SureScan MRI, Medtronic) and a nonrechargeable model (PrimeAdvanced SureScan MRI, Medtronic) were used. Any excess electrode lead was coiled behind the IPG and secured with anchoring sutures to the fascia before layered wound closure.

For patients requiring surgical leads, the procedure was performed under general anesthesia with endotracheal intubation. Using intraoperative fluoroscopy, the targeted spinal levels were marked, and a midline incision was made. The paraspinal muscles were dissected subperiosteally, and the interspinous ligament and ligamentum flavum were removed to expose the epidural space. A surgical lead was then advanced in a caudorostral direction and positioned under fluoroscopic guidance. After confirming the SCS system’s connectivity and impedance, the leads were anchored and a strain relief loop was created. The leads were then tunneled to a subcutaneous IPG pocket, and the IPG was implanted as described above. The skin was closed in layers.

On postoperative day one, stimulus parameters and program settings were optimized. Patients were discharged once satisfactory pain coverage and device stability were confirmed. Routine wound care was performed, and long-term follow-up was scheduled to monitor SCS outcomes.

### Data collection and outcome assessment

Clinical and outcome data were systematically collected from each participant. Clinical characteristics included sex, age at the time of SCS, and the duration of neuropathic pain from its onset to the date of the trial procedure. Pain origin was classified as peripheral nerve or spinal cord. Peripheral nerve origin encompassed primary lesions in the peripheral nerve, spinal nerve, nerve root, or cauda equina. Spinal cord origin referred to a primary lesion within the spinal cord. Etiology was categorized as traumatic or non-traumatic, and pain distribution was classified as localized if it involved only a few consecutive dermatomes (less than one limb), or regional if it involved at least one limb or a more extensive area.

Pain intensity was assessed using the numeric pain rating score (NPRS) at three time points: before the trial procedure (baseline), at the end of the trial phase, and during long-term follow-up after permanent stimulator implantation. Trial outcomes were defined as either positive (≥ 50% reduction in NPRS) or negative (< 50% reduction in NPRS) compared with the baseline. Following permanent implantation, SCS outcomes were classified as favorable (≥ 70% reduction in NPRS) or unfavorable (< 70% reduction in NPRS) compared with the baseline. Any adverse effects related to the SCS procedure were also documented.

### Statistical analysis

IBM SPSS Statistics 24.0 (IBM Corp, Armonk, NY, USA) was used for data analysis. Descriptive statistics were employed for both qualitative and quantitative data, and the Kolmogorov‒Smirnov test was used to assess normality of quantitative variables. The Mann‒Whitney *U* test or the independent *t*-test was applied to compare quantitative data between two independent groups.

For categorical data, Pearson’s chi-square or Fisher’s exact test was used to explore the association between variables and compare outcomes between the groups. The strength of association was reported as the odds ratio (OR) with a 95% confidence interval (CI). A *p* value < 0.05 was considered statistically significant.

## Results

A total of 27 patients met the inclusion criteria. Their mean age was 49.6 ± 14.1 years, with 13 males (48.1%) and 14 females (51.9%). The mean interval between neuropathic pain onset and the SCS procedure was 51 months (range 6‒110). Thirteen patients (48.1%) had localized pain distribution, whereas 14 (51.9%) presented with regional pain. Sixteen out of twenty-seven cases used dual electrode leads during the trial stimulation. The primary reasons for utilizing dual leads consisted of pain distribution bilaterally, involvement of the paramedian area, or unilateral pain with significant dermatomal spread.

Seventeen patients (63%) demonstrated a positive trial stimulation, while the remaining 10 (37%) had a negative trial. The overall median improvement in the NPRS was 58.6% compared with the preoperative baseline. Of the 17 patients with a positive trial, one declined permanent SCS implantation. Thus, 16 patients proceeded to SCS implantation, achieving a median NPRS improvement of 72.5% compared with baseline. Concerning electrode lead types, the 8-contact percutaneous, 2 × 8 surgical, and 5–6-5 surgical leads were used in 8, 3, and 5 patients, respectively. The median postoperative follow-up interval among these 16 patients was 34 months (range 12‒89). A summary of clinical characteristics and treatment outcomes is shown in Table 1.

**Table 1:** Clinical characteristics and treatment outcomes in all patients (*n* = 27)

Total number of cases, *n* (%)		27 (100)
Sex	Male, n (%)	13 (48.1)
	Female, n (%)	14 (51.9)
Age at surgery (years), mean ± SD		49.6 ± 14.1
Duration between pain onset and SCS procedure (months), median (range)		51 (6–110)
Pain distribution	Localized^a^, n (%)	13 (48.1)
	Regional^b^, n (%)	14 (51.9)
Cause of pain, n (%)		
Peripheral nerve origin (*n* = 14)		
Peripheral nerve lesion	Brachial plexus injury	2 (7.4)
	Brachial plexitis	1 (3.7)
	Post-surgical intercostal neuralgia	2 (7.4)
	Post-herpetic neuralgia	1 (3.7)
	Post-surgical schwannoma	1 (3.7)
Spinal nerve or nerve root lesion	Post-surgical schwannoma	4 (14.8)
	Cauda equina injury	2 (7.4)
	Post-surgical Tarlov’s cyst	1 (3.7)
Spinal cord origin (*n* = 13)		
Non-traumatic spinal cord lesion	Spinal cord tumor	3 (11.1)
	Spinal cord cavernoma	1 (3.7)
	Syringomyelia-associated Chiari I malformation	4 (14.8)
Traumatic spinal cord lesion	Spinal cord injury	5 (18.6)
Spinal cord stimulator trial	Positive^c^, n (%)	^g^17 (63)
	Negative^d^, n (%)	10 (37)
Long-term change in postoperative numeric pain rating score^e^ (%), median (range)		+ 58.6 (–14.3 to + 100)
Postoperative follow-up^f^ (months), median (range)		34 (12–89)

n, number of patients; SCS, spinal cord stimulation; SD, standard deviation^a^Localized neuropathic pain involved a few consecutive dermatomes but affected less than one limb^b^Regional neuropathic pain involved one or more limbs^c^Positive trial: ≥ 50% reduction in numeric pain rating score (NPRS) during the trial phase compared with the preoperative score^d^Negative trial: < 50% reduction in NPRS during the trial phase compared with the preoperative score^e^Percentage change in NPRS after SCS compared with preoperative baseline. A positive value (+) indicates pain improvement, while a negative value (–) indicates pain worsening^f^Postoperative follow-up period in the 16 patients who underwent SCS implantation after a positive trial^g^One patient with a positive trial declined permanent implantation of the spinal cord stimulator

When comparing peripheral nerve and spinal cord origins of neuropathic pain (Table 2), patients with peripheral nerve lesions were more likely to have localized pain (*p* = 0.012, OR = 8.33, 95% CI = 1.47‒47.23). This group also showed higher rates of positive stimulation trials (*p* = 0.018, OR = 9.60, 95% CI = 1.48‒62.16) and demonstrated significantly greater postprocedural NPRS improvement (median improvement: 68.4% vs. 10.6%, *p* = 0.006).

**Table 2:** Comparison of clinical characteristics and treatment outcomes between patients with neuropathic pain of peripheral nerve and spinal cord origins (*n* = 27)

	Neuropathic pain of peripheral nerve origin (*n* = 14)	Neuropathic pain of spinal cord origin (*n* = 13)	*p* value	OR (95% CI)
Sex, n (%)			0.332	
Male	8 (57.1)	5 (38.5)		2.13 (0.46–9.94)
Female	6 (42.9)	8 (61.5)		1.00
Age (years), mean ± SD	48.6 ± 15.5	50.7 ± 13.1	0.715	
Duration between pain onset and SCS procedure (months), median (range)	43 (6–110)	54 (29–98)	0.322	
Pain distribution, n (%)			0.012^a^	
Localized^b^	10 (71.4)	3 (23.1)		8.33 (1.47–47.23)
Regional^c^	4 (28.6)	10 (76.9)		1.00
Spinal cord stimulator trial, n (%)			0.018^a^	
Positive^d^	^g^12 (85.7)	5 (38.5)		9.60 (1.48–62.16)
Negative^e^	2 (14.3)	8 (61.5)		1.00
Long-term change in postoperative numeric pain rating score^f^ (%), median (range)	+ 68.4 (0 to + 100)	+ 10.6 (–14.3 to + 100)	0.006^a^	

CI, confidence interval; n, number of patients; OR, odds ratio; SCS, spinal cord stimulation; SD, standard deviation^a^Indicates statistical significance^b^Localized neuropathic pain involved a few consecutive dermatomes but affected less than one limb^c^Regional neuropathic pain involved one or more limbs^d^Positive trial: ≥ 50% reduction in numeric pain rating score (NPRS) during the trial phase compared with the preoperative score^e^Negative trial: < 50% reduction in NPRS during the trial phase compared with the preoperative score^f^Percentage change in NPRS after SCS compared with the preoperative score; a positive value (+) indicates pain improvement, and a negative value (–) indicates worsening of pain^g^One patient with a positive trial declined permanent implantation of the spinal cord stimulator

Regarding etiology (Table 3), male sex was strongly associated with traumatic pain (*p* = 0.018, OR = 9.6, 95% CI = 1.48‒62.16). No other variables showed a significant relationship with etiology in terms of trial stimulation outcomes or postoperative pain relief.

**Table 3:** Comparison of clinical characteristics and treatment outcomes between patients with neuropathic pain of traumatic and non-traumatic etiologies (*n* = 27)

	Neuropathic pain of traumatic etiology (*n* = 10)	Neuropathic pain of non-traumatic etiology (*n* = 17)	*p* value	OR (95% CI)
Sex, n (%)			0.018^a^	
Male	8 (80)	5 (29.4)		9.60 (1.48–62.16)
Female	2 (20)	12 (70.6)		1.00
Age (years), mean ± SD	48.6 ± 17.3	50.2 ± 12.5	0.778	
Duration between pain onset and SCS procedure (months), median (range)	49.5 (15–110)	51 (6–98)	0.780	
Pain distribution, n (%)			0.695	
Localized^b^	4 (40)	9 (52.9)		0.59 (0.12–2.89)
Regional^c^	6 (60)	8 (47.1)		1.00
Spinal cord stimulator trial, n (%)			0.415	
Positive^d^	5 (50)	^g^12 (70.6)		0.42 (0.08–2.11)
Negative^e^	5 (50)	5 (29.4)		1.00
Long-term change in postoperative numeric pain rating score^f^ (%), median (range)	+ 48.4 (–14.3 to + 90)	+ 57.1 (–14.3 to + 100)	0.897	

CI, confidence interval; n, number of patients; OR, odds ratio; SCS, spinal cord stimulation; SD, standard deviation^a^Indicates statistical significance.^b^Localized neuropathic pain involved a few consecutive dermatomes but affected less than one limb.^c^Regional neuropathic pain involved one or more limbs.^d^Positive trial: ≥ 50% reduction in numeric pain rating score (NPRS) during the trial phase compared with the preoperative score.^e^Negative trial: < 50% reduction in NPRS during the trial phase compared with the preoperative score.^f^Percentage change in NPRS after SCS compared with the preoperative score. A positive value (+) indicates pain improvement, and a negative value (–) indicates pain worsening.^g^One patient with a positive trial declined permanent implantation of the spinal cord stimulator.

Analysis of variables associated with a positive SCS trial (Table 4) reinforced that peripheral nerve origin was a significant factor (*p* = 0.018, OR = 9.60, 95% CI = 1.48‒62.16). During the trial phase, patients with a positive trial experienced a median NPRS improvement of 70% compared with 5% in those with a negative trial (*p* < 0.001).

**Table 4:** Comparison of clinical characteristics and treatment outcomes between groups with positive and negative spinal cord stimulator trials (*n* = 27)

	Spinal cord stimulation trial		*p* value	OR (95% CI)
	Positive^b^ (*n* = 17^g^)	Negative^c^ (*n* = 10)		
Sex, n (%)			0.257	
Male	6 (35.3)	6 (60)		0.36 (0.07–1.82)
Female	11 (64.7)	4 (40)		1.00
Age (years), mean ± SD	48.9 ± 12.8	51.4 ± 16.1	0.689	
Duration between pain onset and SCS procedure (months), median (range)	49 (15–110)	52.5 (6–98)	0.897	
Pain distribution, n (%)			0.107	
Localized^d^	10 (58.8)	2 (20)		5.71 (0.92–35.48)
Regional^e^	7 (41.2)	8 (80)		1.00
Pain origin, n (%)			0.018^a^	
Peripheral nerve	12 (70.6)	2 (20)		9.60 (1.48–62.16)
Spinal cord	5 (29.4)	8 (80)		1.00
Pain etiology, n (%)			0.415	
Traumatic	5 (29.4)	5 (50)		0.42 (0.08–2.11)
Non-traumatic	12 (70.6)	5 (50)		1.00
Change in numeric pain rating score during the trial phase^f^ (%), median (range)	+ 70 (–14.3 to + 100)	+ 5 (–14.3 to + 33.3)	< 0.001^a^	

CI, confidence interval; n, number of patients; OR, odds ratio; SCS, spinal cord stimulation; SD, standard deviation^a^Indicates statistical significance.^b^Positive trial: ≥ 50% reduction in numeric pain rating score (NPRS) during the trial phase compared with the preoperative score.^c^Negative trial: < 50% reduction in NPRS during the trial phase compared with the preoperative score.^d^Localized neuropathic pain involved a few consecutive dermatomes but affected less than one limb.^e^Regional neuropathic pain involved one or more limbs.^f^Percentage change in NPRS during the trial phase compared with the preoperative score; a positive value (+) indicates pain improvement, and a negative value (–) indicates pain worsening.^g^One patient with a positive trial declined permanent implantation of the spinal cord stimulator.

After permanent implantation, no variable emerged as significantly related to a favorable outcome (≥ 70% reduction in NPRS; Table 5). However, median NPRS improvement in the favorable-outcome group (85.7%) surpassed that in the unfavorable-outcome group (60%; *p* = 0.001).

**Table 5:** Comparison of clinical characteristics and treatment outcomes between patients with favorable and unfavorable outcomes following spinal cord stimulator implantation (*n* = 16)

	Treatment outcome		*p* value	OR (95% CI)
	Favorable^b^ (*n* = 9)	Unfavorable^c^ (*n* = 7)		
Sex, n (%)			0.633	
Male	4 (44.4)	2 (28.6)		0.50 (0.06–4.09)
Female	5 (55.6)	5 (71.4)		1.00
Age (years), mean ± SD	51.1 ± 15.9	45.9 ± 9.1	0.490	
Duration between pain onset and SCS procedure (months), median (range)	38 (15–75)	64 (31–110)	0.395	
Pain distribution, n (%)			1.000	
Localized^d^	6 (66.7)	4 (57.1)		1.50 (0.20–11.54)
Regional^e^	3 (33.3)	3 (42.9)		1.00
Pain origin, n (%)			0.596	
Peripheral nerve	7 (77.8)	4 (57.1)		2.63 (0.30–23)
Spinal cord	2 (22.2)	3 (42.9)		1.00
Pain etiology, n (%)			1.000	
Traumatic	3 (33.3)	2 (28.6)		1.25 (0.15–10.7)
Non-traumatic	6 (66.7)	5 (71.4)		1.00
Long-term change in postoperative numeric pain rating score^f^ (%), median (range)	+ 85.7 (+ 70 to + 100)	+ 60 (–14.3 to 66.7)	0.001^a^	

CI, confidence interval; n, number of patients; OR, odds ratio; SCS, spinal cord stimulation; SD, standard deviation^a^Indicates statistical significance^b^Favorable outcome: ≥ 70% reduction in numeric pain rating score (NPRS) after SCS implantation compared with the preoperative score^c^Unfavorable outcome: < 70% reduction in NPRS after SCS implantation compared with the preoperative score^d^Localized neuropathic pain involved a few consecutive dermatomes but affected less than one limb^e^Regional neuropathic pain involved one or more limbs^f^Percentage change in NPRS after SCS implantation compared with the preoperative score; a positive value (+) indicates pain improvement, and a negative value (–) indicates pain worsening.

No surgery-related or hardware-related complications were observed. None of the patients developed electrode migration, device infection, or new neurological deficits. A few individuals reported stimulation-related side effects, such as painful paresthesias or incomplete coverage of the painful area, but these issues were corrected through precise adjustments of stimulus parameters.

## Discussion

Neuropathic pain is characterized as pain that arises from an injury impacting the nervous system. It is marked by burning, electric shock-like, or stabbing features. The pain occurs spontaneously or in association with stimuli, like allodynia or hyperalgesia. Managing neuropathic pain in everyday practice is challenging. Treatment outcomes can be influenced by a range of factors, including the choice of medication, response to pharmacotherapy, and patient phenotyping [23].

Even with appropriate treatment, a subset of patients develops persistent or refractory neuropathic pain [24]. Refractory pain correlates with increased disability, poor quality of life, and high healthcare utilization [25]. In such cases, SCS is considered a viable option for patients who fail to respond to standard therapies. It often serves as a final resort for achieving adequate pain relief.

SCS is recognized as effective for various types of neuropathic pain [26, 27]. It has been shown to benefit peripheral neuropathy, post-herpetic neuralgia, diabetic neuropathy, brachial plexus injuries, SCIs or lesions, and even cancer pain [19, 27–37]. This study compared outcomes of SCS for neuropathic pain originating in the peripheral nerve versus the spinal cord. We observed that localized pain was more common in peripheral nerve lesions, whereas regional neuropathic pain was predominant in spinal cord lesions.

Peripheral nerves encompass all neural structures outside the brain and spinal cord [38]. Neuropathic pain stemming from a peripheral nerve, particularly mononeuropathies or radiculopathies, often remains confined to the nerve’s anatomic distribution [39]. In contrast, spinal cord lesions frequently manifest a more extensive distribution of neuropathic pain than peripheral nerve lesions. Patients with SCI often exhibit at-level and below-level neuropathic pain, which are characteristic patterns [40–42].

At-level pain arises from increased expression of N-methyl-D-aspartate and glutamate receptors, altered sodium and calcium channel functions, glial activation, and weakened inhibitory neuronal mechanisms. These changes trigger hyperexcitability of spinal neurons and culminate in neuropathic pain [43]. Below-level pain primarily results from direct spinal cord damage, leading to the disruption of descending inhibitory tracts and loss of normal inhibitory signaling. These processes foster abnormal neuronal firing near the lesion, which contributes to neuropathic pain [44, 45]. Higher-level processes in the brain, including increased thalamic firing and altered thalamic perfusion, may further exacerbate below-level pain [46, 47].

In comparing trial stimulation outcomes, we observed a higher success rate in patients with neuropathic pain of peripheral nerve origin than in those with spinal cord lesions. Likewise, the peripheral nerve origin group achieved significantly better long-term reductions in NPRS. Several factors may explain these findings. First, patients with peripheral nerve lesions often have more localized pain distribution, because their pathology involves the nerve or nerve root rather than the spinal cord [48]. Consequently, generating paresthesia that fully covers the painful area is generally easier in peripheral lesions than in spinal cord lesions, where the pain may be more extensive.

Second, SCS is administered within the epidural space. Many individuals with spinal cord lesions have a history of spine surgery or fixation, leading to epidural granulation or scar tissue. These changes can interfere with optimal electrode positioning and potentially diminish both trial and long-term stimulation efficacy.

Neuropathic pain secondary to SCI remains complex and difficult to manage. Typically, our therapeutic approach involves SCS, dorsal root entry zone lesioning, or a combination of both to address this refractory condition. In our study, five patients with SCI-related neuropathic pain all failed to achieve a positive trial stimulation result, and none proceeded to permanent device implantation. This finding aligns with previous studies suggesting limited efficacy of SCS for SCI-related neuropathic pain [49–51]. For example, a study by Sokal et al. reported that only 2 of 8 SCI patients experienced satisfactory pain relief even with burst and high-frequency SCS [51]. Nevertheless, several reports have demonstrated improvement in neuropathic pain arising from SCI [52–54] or spinal cord lesions [55], particularly when additional modalities such as burst [52] or high-frequency stimulation [54] were used. Similarly, dorsal root entry lesioning has shown poorer outcomes for SCI-related pain compared with pain arising from cauda equina lesions [56]. These collective findings underscore the formidable challenges in treating neuropathic pain in SCI, highlighting the need for novel therapeutic strategies.

No statistically significant difference in trial stimulation outcomes or long-term SCS results emerged between patients with traumatic versus non-traumatic neuropathic pain. Similarly, there was no discrepancy in success rates or sustained pain relief between localized and regional neuropathic pain distributions. Hence, candidates for SCS may include patients with neuropathic pain following traumatic events or those experiencing regional pain patterns involving one or more extremities.

### Strengths and limitations

A major strength of this study was the strict inclusion of individuals with a clearly defined primary lesion in either the peripheral nerve or the spinal cord. By excluding patients with failed back surgery syndrome, failed neck surgery syndrome, and complex regional pain syndrome—conditions commonly associated with multifactorial pain generators—selection bias was reduced. To our knowledge, no prior research has directly compared SCS outcomes between neuropathic pain of peripheral nerve and spinal cord origins.

Several limitations should be noted. First, in our institution, failed back surgery syndrome and complex regional pain syndrome are the most common indications for SCS, whereas other neuropathic etiologies are less prevalent. Consequently, the sample size in this study was relatively small, although certain comparisons still reached statistical significance. Second, we relied exclusively on conventional SCS and did not employ burst or high-frequency stimulation, both of which have been associated with distinct analgesic benefits in SCI [48]. These advanced stimulation paradigms might enhance outcomes in patients with spinal cord lesions. Third, we focused on changes in pain intensity rather than on quality-of-life measures. In our view, greater neurological sequelae in SCI could confound comparisons of daily functioning and overall quality of life with those seen in peripheral nerve lesions. Consequently, comparing quality-of-life measures between these two groups may not yield meaningful insights due to their fundamentally different degrees of neurological impairment.

Ultimately, a comparatively low ratio of trial to permanent device implantation was observed in our study. Out of 27 patients, 16 received permanent device implantation. The ratio of trials to implantations was 0.59, indicating it was quite low. When it comes to effective pain management, the response of chronic neuropathic pain to SCS was inconsistent compared to groups with typical indications for SCS, such as failed back surgery syndrome, complex regional pain syndrome, and peripheral vascular disease. Saengsomsuan et al. conducted a prospective study to assess the long-term effects of SCS between groups with usual (failed back surgery syndrome, complex regional pain syndrome, and peripheral vascular disease) and unusual indications (chronic neuropathic pain from different causes, phantom limb pain) for SCS. The implant to trial ratio was notably greater in the usual indications (ratio of 0.77) compared to unusual indications (ratio of 0.40) for SCS (*p* = 0.016, odds ratio = 5.14). Additionally, patients with usual indications experienced greater improvement and more consistency in pain intensity, pain-related interference, and health-related quality of life (HRQOL) than those with unusual indications [22].

## Conclusions

SCS is a feasible and effective treatment for chronic refractory neuropathic pain arising from peripheral nerve or spinal cord lesions. Patients with peripheral nerve involvement demonstrated higher rates of trial success and superior long-term outcomes than those with spinal cord lesions. In particular, patients with traumatic SCIs yielded the poorest results. These findings may aid clinicians in selecting appropriate candidates for SCS and estimating likely treatment outcomes.

## Data Availability

No datasets were generated or analysed during the current study.
